# Enhancement of potent immune responses to HPV16 E7 antigen by using different vaccine modalities

**Published:** 2011-01-10

**Authors:** Azam Bolhassani, Elham Mohit, Nahid Ghasemi, Maryam Salehi, Mohammad Taghikhani, Sima Rafati

**Affiliations:** 1 Molecular Immunology and Vaccine Research Laboratory, Pasteur Institute of Iran, Tehran, Iran; 2 Department of Clinical Biochemistry, Tarbiat Modarres University, Tehran, Iran; 3 Department of Immunology, Shahid Beheshti University, Tehran, Iran

## 

Human papillomaviruses (HPVs) are responsible for an enormous global burden of genital disease. HPV is annually associated with 500,000 new cases of cervical cancer and 250,000 cervical cancer deaths worldwide. There are more than 130 HPV genotypes that have been recognized from various clinical lesions. HPV types 16 and 18 are found in the majority of cervical cancer cases. The association between HPV infection and cervical cancer indicates that HPV serves as an ideal target for development of preventive and therapeutic vaccines. HPV genome codes eight early or regulatory proteins (E1-E8) and two late or capsid proteins (L1/L2). Two HPV oncogenic proteins, E6 and E7, are consistently co-expressed in HPV-expressing cervical cancers and are important in the induction and maintenance of cellular transformation. Therefore, immunotherapy targeting E6 and/or E7 proteins provides an opportunity to prevent and treat HPV-associated cervical malignancies. Effective therapeutic HPV vaccines should generate strong E6/E7-specific T cell-mediated immune responses. Currently, we have focused on different vaccine modalities including DNA vaccines, protein vaccines, live vaccines and the combined approaches (e.g., prime-boost vaccines) against HPV infections. In our studies, various strategies were applied to enhance DNA vaccine potency including the utilization of adjuvants especially heat shock proteins (e.g., GP96) and delivery systems such as polyethyleneimine (PEI), polymer-peptide hybrid (PEI600-Tat conjugate) and also electroporation. At first, the level of humoral and cellular immune responses were compared by using HPV16 E7 + Gp96 co-injection as DNA/DNA and prime-boost (DNA/ protein) immunization strategies in C57BL/6 mice model. Assessment of cellular immune responses against E7 antigen indicated that co-delivery of naked DNA E7 + Gp96 plasmid is immunologically more effective than E7 alone and induces Th1 response [1,2].

Furthermore, in another study we demonstrated that PEI600-Tat conjugate is efficient to improve immune responses *in vivo*. Indeed, PEI600-Tat/E7DNA complex at certain ratio induced IFN-γ response slightly more than that GP96 adjuvant [3]. In our recent study, we are attempting to develop a novel, non-pathogenic, parasitic vector, *Leishmania tarentolae*, as a recombinant live HPV vaccine candidate expressing HPV16 E7 and evaluate its ability to elicit E7-specific T cell responses in mice model as compared to E7 DNA vaccine. Moreover, a preventive and therapeutic DNA vaccine has been generated using HPV16 E7 co-linked to N-terminal fragment of GP96 and chemokines delivered by chemical system (PEI600-Tat conjugate) and/or physical method (electroporation). Altogether, combining new and improved immunotherapeutic strategies are crucial for enhancing long-term immune responses in patients.
